# Supplementary material for: The Japanese Guide affected the prescription of steroids for COVID-19 inpatients during the COVID-19 epidemic in Japan
Source: Sci Rep. 2023 Jun 3;13:9041. doi: 10.1038/s41598-023-36199-w (PMC10239045; doi:10.1038/s41598-023-36199-w)
Supplement: Supplementary file 1 — Supplementary Figure 1. [file 41598_2023_36199_MOESM1_ESM.pptx]

## Slide 1
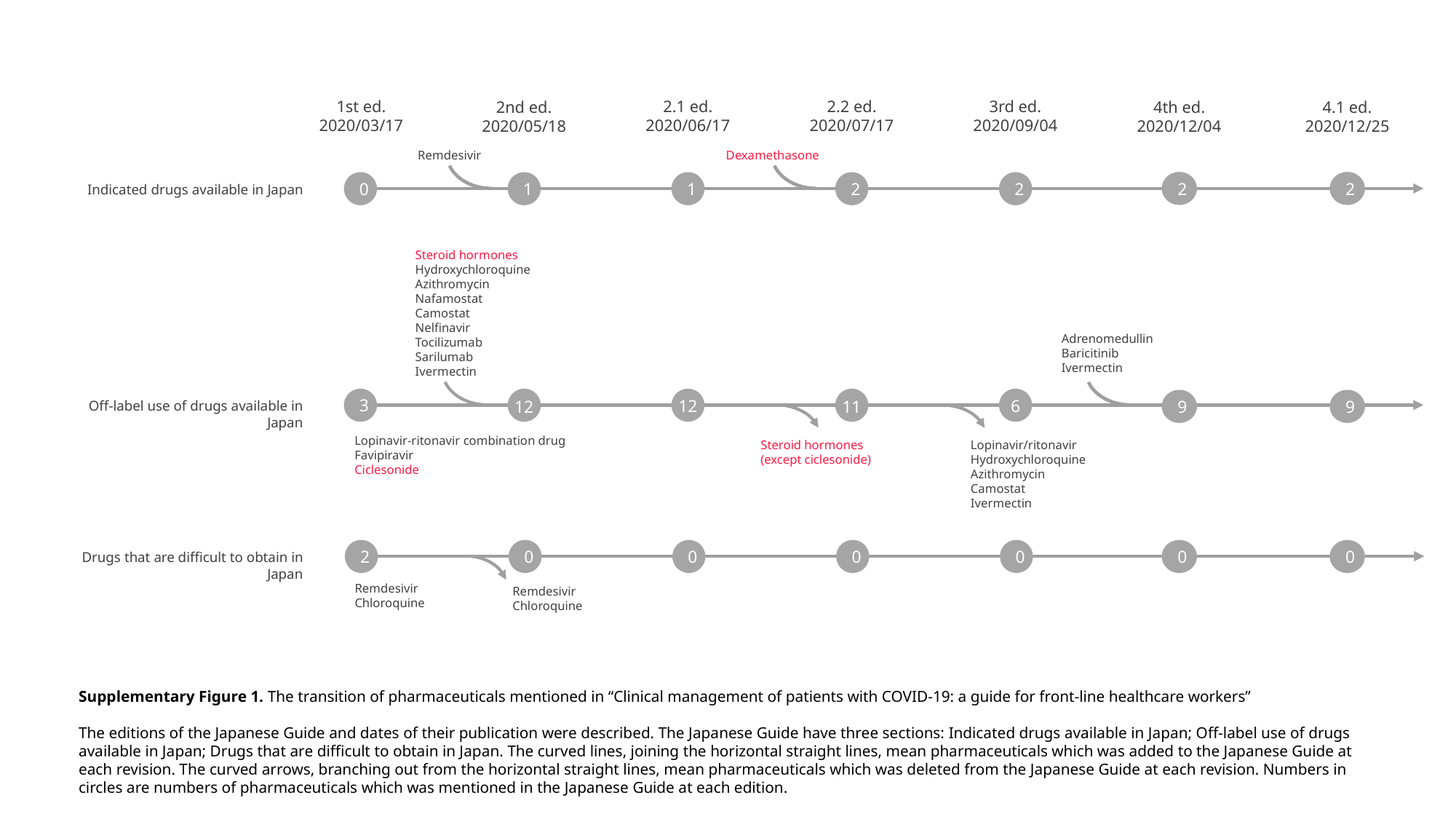

1st ed.
2020/03/17
2.1 ed.
2020/06/17
2.2 ed.
2020/07/17
3rd ed.
2020/09/04
4th ed.
2020/12/04
4.1 ed.
2020/12/25
2nd ed.
2020/05/18
Dexamethasone
Remdesivir
2
2
0
1
1
2
2
Indicated drugs available in Japan
Steroid hormones
Hydroxychloroquine
Azithromycin
Nafamostat
Camostat
Nelfinavir
Tocilizumab
Sarilumab
Ivermectin
Adrenomedullin
Baricitinib
Ivermectin
3
9
9
12
6
12
11
Off-label use of drugs available in Japan
Lopinavir-ritonavir combination drug
Favipiravir
Ciclesonide
Steroid hormones
(except ciclesonide)
Lopinavir/ritonavir
Hydroxychloroquine
Azithromycin
Camostat
Ivermectin
2
0
0
0
0
0
0
Drugs that are difficult to obtain in Japan
Remdesivir
Chloroquine
Remdesivir
Chloroquine
Supplementary Figure 1. The transition of pharmaceuticals mentioned in “Clinical management of patients with COVID-19: a guide for front-line healthcare workers”
The editions of the Japanese Guide and dates of their publication were described. The Japanese Guide have three sections: Indicated drugs available in Japan; Off-label use of drugs available in Japan; Drugs that are difficult to obtain in Japan. The curved lines, joining the horizontal straight lines, mean pharmaceuticals which was added to the Japanese Guide at each revision. The curved arrows, branching out from the horizontal straight lines, mean pharmaceuticals which was deleted from the Japanese Guide at each revision. Numbers in circles are numbers of pharmaceuticals which was mentioned in the Japanese Guide at each edition.
