# Supplementary material for: The Japanese Guide affected the prescription of steroids for COVID-19 inpatients during the COVID-19 epidemic in Japan
Source: Sci Rep. 2023 Jun 3;13:9041. doi: 10.1038/s41598-023-36199-w (PMC10239045; doi:10.1038/s41598-023-36199-w)
Supplement: Supplementary file 2 — Supplementary Table 1. [file 41598_2023_36199_MOESM2_ESM.pptx]

## Slide 1
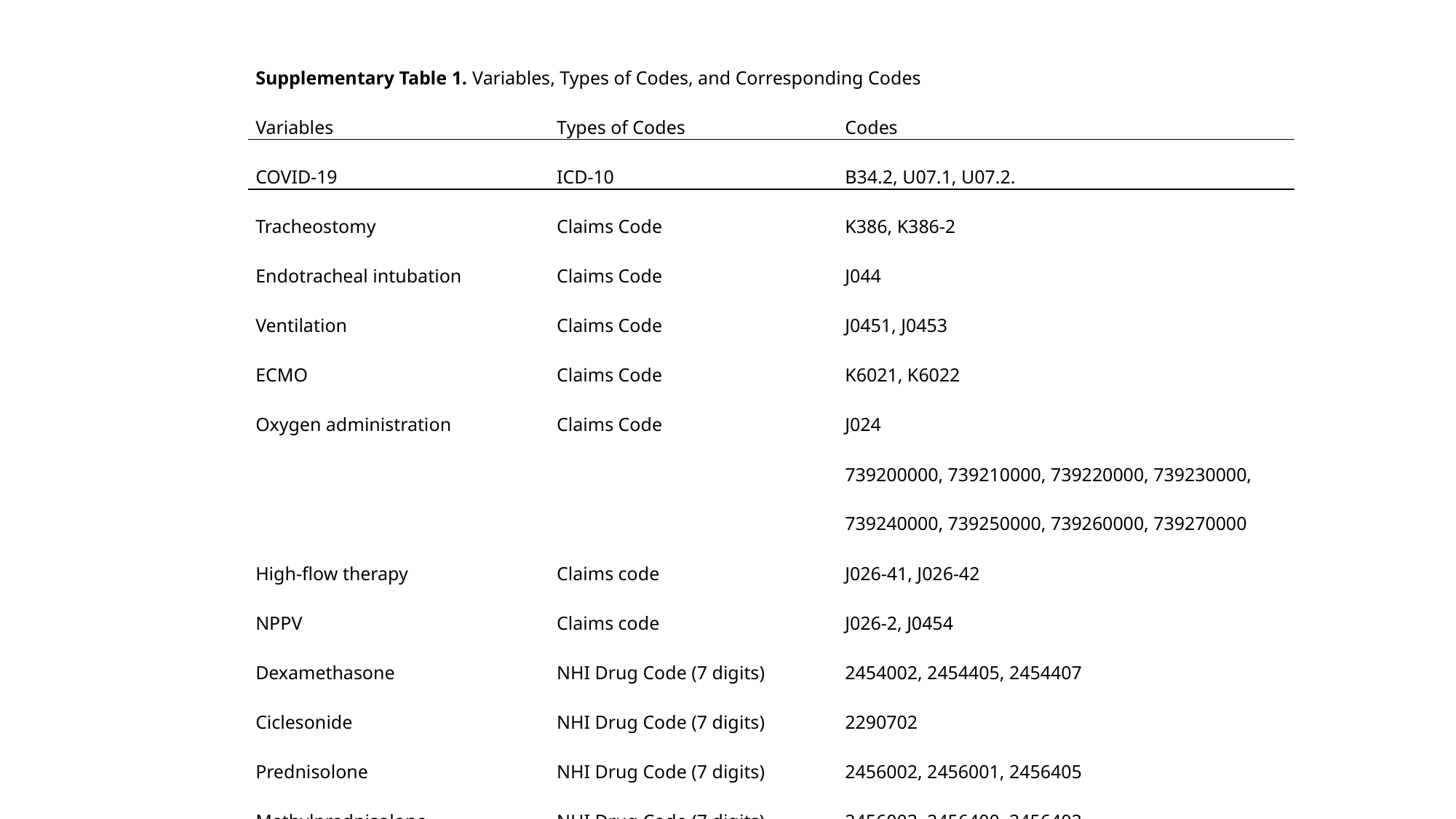

| Supplementary Table 1. Variables, Types of Codes, and Corresponding Codes | | |
| --- | --- | --- |
| Variables | Types of Codes | Codes |
| COVID-19 | ICD-10 | B34.2, U07.1, U07.2. |
| Tracheostomy | Claims Code | K386, K386-2 |
| Endotracheal intubation | Claims Code | J044 |
| Ventilation | Claims Code | J0451, J0453 |
| ECMO | Claims Code | K6021, K6022 |
| Oxygen administration | Claims Code | J024 |
| | | 739200000, 739210000, 739220000, 739230000, 739240000, 739250000, 739260000, 739270000 |
| High-flow therapy | Claims code | J026-41, J026-42 |
| NPPV | Claims code | J026-2, J0454 |
| Dexamethasone | NHI Drug Code (7 digits) | 2454002, 2454405, 2454407 |
| Ciclesonide | NHI Drug Code (7 digits) | 2290702 |
| Prednisolone | NHI Drug Code (7 digits) | 2456002, 2456001, 2456405 |
| Methylprednisolone | NHI Drug Code (7 digits) | 2456003, 2456400, 2456402 |
| Abbreviations: COVID-19, Coronavirus disease 2019; ICD-10, International Statistical Classification of Diseases and Related Health Problems, Tenth Revision; ECMO, Extracorporeal Membrane Oxygenation; NPPV, Non invasive Positive Pressure Ventilation; NHI, National Health Insurance; | | |
